# Supplementation with cod protein hydrolysate in older adults: a dose range cross-over study

**DOI:** 10.1017/jns.2019.37

**Published:** 2019-12-04

**Authors:** Caroline Jensen, Hanna F. Dale, Trygve Hausken, Einar Lied, Jan G. Hatlebakk, Ingeborg Brønstad, Gülen A. Lied, Dag Arne L. Hoff

**Affiliations:** 1 Department of Clinical Medicine, Centre for Nutrition, University of Bergen, Bergen, Norway; 2 Division of Gastroenterology, Department of Medicine, Haukeland University Hospital, Bergen, Norway; 3 National Centre of Functional Gastrointestinal Disorders, Haukeland University Hospital, Bergen, Norway; 4 Firmenich Bjørge Biomarin AS, Ellingsøy, Ålesund, Norway; 5 National Centre for Ultrasound in Gastroenterology, Haukeland University Hospital, Bergen, Norway; 6 Division of Gastroenterology, Department of Medicine, Ålesund Hospital, Møre & Romsdal Hospital Trust, Ålesund, Norway; 7 Department of Clinical and Molecular Medicine, Faculty of Medicine and Health Sciences, Norwegian University of Science and Technology, Trondheim, Norway

**Keywords:** Fish protein, Cod protein, Marine peptides, Marine protein hydrolysate, Glucose homeostasis, BW, body weight, CPH, cod protein hydrolysate, GLP-1, glucagon-like peptide 1

## Abstract

A large proportion of older adults are affected by impaired glucose metabolism. Previous studies with fish protein have reported improved glucose regulation in healthy adults, but the evidence in older adults is limited. Therefore, we wanted to assess the effect of increasing doses of a cod protein hydrolysate (CPH) on postprandial glucose metabolism in older adults. The study was a double-blind cross-over trial. Participants received four different doses (10, 20, 30 or 40 mg/kg body weight (BW)) of CPH daily for 1 week with 1-week washout periods in between. The primary outcome was postprandial response in glucose metabolism, measured by samples of serum glucose and insulin in 20 min intervals for 120 min. The secondary outcome was postprandial response in plasma glucagon-like peptide 1 (GLP-1). Thirty-one subjects aged 60–78 years were included in the study. In a mixed-model statistical analysis, no differences in estimated maximum value of glucose, insulin or GLP-1 were observed when comparing the lowest dose of CPH (10 mg/kg BW) with the higher doses (20, 30 or 40 mg/kg BW). The estimated maximum value of glucose was on average 0·28 mmol/l lower when the participants were given 40 mg/kg BW CPH compared with 10 mg/kg BW (*P* = 0·13). The estimated maximum value of insulin was on average 5·14 mIU/l lower with 40 mg/kg BW of CPH compared with 10 mg/kg BW (*P* = 0·20). Our findings suggest that serum glucose and insulin levels tend to decrease with increasing amounts of CPH. Due to preliminary findings, the results require further investigation.

The human body is dependent on a tight regulation of blood glucose levels to ensure normal function^(1)^. Blood glucose levels are regulated within a narrow range, and glucose homeostasis is maintained through an intricate network of hormones and neuropeptides that are released in the body^(1,2)^. With increasing age, glucose metabolism changes and a large proportion of older adults are affected by impaired glucose metabolism^(3,4)^. Since skeletal muscle is the major site for insulin-stimulated uptake of glucose^(5,6)^, it has been suggested that low skeletal muscle mass observed in some older adults with reduced muscle mass and function might result in reduced capacity for glucose disposal^(7)^. Furthermore, higher fasting and postprandial values of glucose and insulin have been associated with lower muscle mass in older adults^(8)^. The gradual decline in muscle mass and function observed with increasing age^(9–11)^ is a major threat to healthy ageing, and causes reduced mobility, increased disability, loss of independence and overall reduced quality of life^(12,13)^.

Several previous intervention studies have reported improved insulin sensitivity^(14,15)^ and glucose tolerance^(14,16)^ in humans and rodents after supplementation with fish protein. Furthermore, 3-month supplementation with a daily dose of 1·4 g protein hydrolysate from blue whiting given to overweight adults increased blood concentrations of glucagon-like peptide 1 (GLP-1). No further effects were observed when the participants were given a higher dose of 2·8 g, which might indicate a plateau effect starting at 1·4 g^(17)^. GLP-1 is released from the enteroendocrine L-cells in response to food intake and lowers blood glucose levels by stimulating insulin secretion, suppressing glucagon secretion and slowing gastric emptying^(18)^. In general, fish protein and hydrolysates from fish protein have a well-balanced distribution of amino acids and should be considered a high-quality protein source, and there is an increasing amount of evidence supporting a favourable effect of these proteins on metabolic health^(19)^.

The evidence of health effects of cod protein as a nutritional supplement is limited, and only a few studies in healthy and overweight adults have been conducted. A recent study reported that an 8-week supplementation with 6 g of residual material from cod (press-cake meal) in a group of overweight or obese adults resulted in decreased postprandial concentrations of serum NEFA, which might indicate an effect on markers for glucose regulation^(20)^. In addition, a small pilot study in overweight adults observed improved glucose regulation after daily supplementation with 2·5 g of protein from cod for 8 weeks^(21)^. No changes in insulin, insulin C-peptide or NEFA in serum were observed^(21)^. Furthermore, we recently demonstrated that supplementation with a single dose of 20 mg/kg body weight (BW) of a protein hydrolysate from cod, given before a breakfast meal, reduced postprandial insulin secretion in forty-one healthy adults between 41 and 64 years, when compared with control^(22)^. We did not observe any effects on postprandial blood glucose response or on the levels of GLP-1.

Based on current knowledge, it is of interest to further explore potential favourable effects of cod protein on parameters closely related to muscle health, including parameters of glucose metabolism in an older population. To our knowledge, no previous trial has evaluated the effect of increasing doses of a supplement with cod protein hydrolysate (CPH) on glucose metabolism in older adults. Therefore, the aim of the present study was to investigate the effect of supplementation with four different weight-adjusted doses of a CPH on postprandial glucose regulation in a group of older adults aged 60–80 years. Based on the results from the study, we hoped to create a basis for selecting an effective daily dose of CPH for further use in clinical study protocols in patient groups with muscle health issues, inflammatory conditions or abnormal glucose metabolism.

## Experimental methods

### Study design

The study was a double-blind cross-over trial. The participants received four different doses (10, 20, 30 or 40 mg/kg BW) of CPH daily for 1 week with 1-week washout periods in between the dose intervals. Each participant received all four different dose intervals in random order. The participants were instructed to take the supplement each morning before breakfast for 7 d. After an initial screening visit, included participants came to the research unit on four different occasions, separated by 2 weeks. In total, the study lasted for 7 weeks.

The primary outcome was postprandial response in glucose metabolism, measured by venous samples of glucose and insulin. Secondary outcomes were plasma GLP-1 and adverse effects measured by symptom questionnaires.

The study was conducted according to the guidelines laid down in the Declaration of Helsinki and all procedures involving human subjects were approved by the Regional Committee for Medical and Health Research Ethics of Central Norway (2017/1795). Written informed consent was obtained from all subjects. The trial is registered at www.clinicaltrials.gov as NCT03526744.

### Participants

Participants were recruited by advertisement on the external websites and on notice boards at Haukeland University Hospital, Bergen and Ålesund Hospital, Ålesund. Recruitment took place between March and July 2018, and the study was conducted between April and November of the same year.

Potential participants were screened for general eligibility by telephone, and suitable candidates were invited for a baseline evaluation visit, with further information and baseline blood chemistry. The criteria for inclusion were age between 60 and 80 years, BMI between 20 and 30 kg/m^2^ and signed informed consent. Criteria for exclusion were allergy and intolerances to fish and/or shellfish, pharmacologically treated diabetes mellitus, low or unstable blood pressure, chronic diseases or medication that were likely to interfere with the evaluation of the study endpoints, acute infections, substance misuse (excessive alcohol consumption and/or narcotic substances assessed by physician) or unwillingness to comply with the requirements of the study. The participants were instructed to not take any nutritional supplements containing *n*-3 PUFA for 2 weeks before study commencement and during the course of the study.

### Study protocol

The participants came to the research unit on five different occasions, including a screening visit. Before inclusion, the subjects underwent clinical examination by a physician, baseline biochemistry and measurement of height, weight and blood pressure.

A 3-d prospective diet diary was recorded prior to starting the intervention, and at the end of the study period. The level of physical activity was assessed at baseline and at the end of the study by asking the participants two questions regarding moderate physical activity and vigorous activity (self-reported). The participants were instructed not to change diet habits or the level of physical activity during the study period.

The study consisted of four different intervention cycles. Before each intervention cycle, the participants received six bottles containing powder with CPH, labelled 1 to 6. We instructed the participants to take one bottle each morning during the intervention cycle. On days of study tests, day 7 in each intervention cycle, the participants came to the research facility in a fasting condition between 08.00 and 09.00 hours. After baseline blood sampling, we gave the last dosage of CPH followed by a standardised breakfast meal 10 min later. At 25 min after the CPH drink was served and 15 min after the breakfast meal had started, we took the first postprandial blood sample (0 min sample). Thereafter, the participants spent 2 h in the vicinity of the research unit to allow for repeated sampling of blood, at 20 min intervals until 120 min.

The standardised breakfast meal consisted of two slices of semi-coarse bread (50 % whole wheat, 80 g bread), 10 g margarine, 20 g strawberry jam and 20 g white cheese, providing a total of 1485 kJ (355 kcal), 41 g carbohydrate, 12·5 g protein and 15 g fat. The drink contained 22·5 g carbohydrate and approximately 418 kJ (100 kcal), and including the drink, the breakfast provided in total 1900 kJ (455 kcal). The amount of energy and carbohydrates in the breakfast was calculated to induce an adequate blood glucose response. No coffee or tea was served, but water was given *ad libitum.*

We handed out the six bottles for the next intervention cycle at the end of the test day and gave instructions for when to start the next intervention cycle. Between intervention cycles, the participants had a washout period of 7 d. All participants received a text message on the morning of the day they were to start the next intervention cycle.

### Assessments

At the screening visit we assessed the participant's medical history and measured biochemical parameters for nutritional status (albumin, prealbumin, vitamins B_12_ and D). We measured biochemical safety parameters at the screening visit and the end of study visit.

During the test days, baseline fasting serum glucose and serum insulin were measured 25 min before the first postprandial blood sample (time (*t*) = 0 min postprandial). Subsequently, serum glucose and insulin were measured every 20 min for 2 h (*t* = 20, 40, 60, 80, 100 and 120 min postprandial). Baseline GLP-1 was measured and thereafter postprandially at *t* = 0, 20, 40, 80 and 120 min. Blood pressure was measured at three time points during the test day as a safety parameter (*t* = 0, 40 and 120 min).

Two questionnaires evaluating the participants’ symptoms were used to identify possible adverse events during each intervention period and on study visits. In each intervention period, a visual analogue scale was filled out before the participants took the first dose with CPH on day 1 and before the last dose on day 7. Further, a questionnaire validated for the evaluation of gastrointestinal symptoms^(23)^ was filled out 2 h after intake of CPH on day 1 and day 7 (end of test day, *t* = 120 min).

### Test material

The protein hydrolysate powder was delivered from the manufacturer (Firmenich Bjørge Biomarin AS) in neutral bottles coded with participant number and dose level (1–4). The bottles were coded by a person not involved in the performance of the study and the different dose levels were randomly allocated to the participants according to a central digital randomisation list. Study participants and investigators were blinded to the dose content in the bottles (double-blinded study). The key of randomisation was provided to the investigators when the trial had ended, and the statistical analysis was completed. The powder contained 4 % protein (CPH raw material) and 96 % carbohydrate (maltodextrin) and was flavoured with lemon. The CPH raw material contained approximately 89 % protein by weight, <0·2 % fat, 0 % carbohydrate, <3·0 % water, 10 % ash, 0·1 % NaCl, 1·7 % Na and 0·07 % chloride. The amino acid composition of CPH raw material is presented in Table 1. The hydrolysation process has been presented in a previous publication^(22)^.

**Table 1.** Amino acid and taurine composition of the cod protein hydrolysate used in the present study

Amino acid	Total amino acid (mg/g)
Alanine	47·8
Arginine	51·1
Aspartic acid	73·3
Asparagine	0·38
Glutamic acid	125·0
Glutamine	0·78
Glycine	50·9
Histidine	13·5
Hydroxyproline	1·0
Isoleucine*	30·1
Leucine*	60·3
Lysine*	71·3
Methionine	22·1
Phenylalanine	23·2
Proline	29·7
Serine	36·0
Taurine	6·6
Threonine	30·9
Tryptophan	6·0
Tyrosine	22·7
Valine	36·9

* Branched-chain amino acids.

### Estimation of energy intake

Calculations of energy and macronutrient intake were performed using *Kostholdsplanleggeren* (Norwegian Food Safety Authority and The Norwegian Directorate of Health, Oslo, Norway)^(24)^, based on the reported food and drink intake data from the participants at baseline and at the end of the study. Participants registered their intake of food and drink for three consecutive days, preferably including one weekend day, prior to the first dose and at the end of the study. The dietary records were used to record the participants’ diet patterns and to assess whether the participants made changes to their diets during the study period.

### Analyses of blood samples

Baseline biochemistry was analysed according to standard accredited methods at the Laboratory for Clinical Biochemistry, Haukeland University Hospital (Bergen, Norway) and the Department of Medical Biochemistry, Ålesund Hospital (Ålesund, Norway).

Glucose and insulin were measured in serum according to standard accredited methods at the Laboratory for Clinical Biochemistry, Haukeland University Hospital (Bergen, Norway). Serum was obtained by centrifugation of full blood at 2000 ***g*** at room temperature (20°C) for 10 min after 30–60 min of coagulation, using serum separator cloth activator tubes. Samples were aliquoted and stored at −80°C prior to analysis.

Plasma for the determination of GLP-1 was obtained by centrifugation of EDTA full blood at 1800 ***g*** at −4°C for 10 min, within 20 min after blood sampling. Prior to sampling, to EDTA blood sampling tubes were added 10 µl dipeptidyl peptidase-4 inhibitor (DPP4-010; DRG Diagnostics) per ml EDTA blood. GLP-1 plasma was aliquoted and stored at −80°C prior to analysis. The GLP-1 analyses were performed using an ELISA kit from IBL International GmbH (GLP-1 (7–36) active ELISA, reference RE53121).

### Statistical analysis

Statistical analyses were performed using Stata v15.1 (StataCorp LLC) and SPSS software (IBM SPSS Statistics 24). Graphical work was conducted in GraphPad Prism version 7.0 (GraphPad Software, Inc.). Data are presented as means and standard deviations for continuous variables, and frequencies and relative frequencies for categorical variables. To estimate the effect of dose we calculated the maximum observed value and the AUC for the time course of each outcome variable, for each combination of person and dose. We then fitted mixed models with the outcome measure (maximum value or AUC) as the dependent variable, fixed effects of dose and random intercepts across persons. Carry-over effects were assessed using a standard likelihood-ratio test to test for interaction between dose and ordering. Paired-samples *t* tests were used to compare changes in energy intake and macronutrient intake from baseline to the end of the study. *P* values <0·05 were considered statistically significant.

The sample size was not feasible to calculate for power analysis, due to lack of similar studies. Possible health effects of supplementation with residual material from cod as protein hydrolysate has previously not been studied in a group of older adults, and therefore we had no basis for calculating sample size. According to protocol, we intended to include thirty participants.

## Results

### Demographic characteristics

**Fig. 1.**
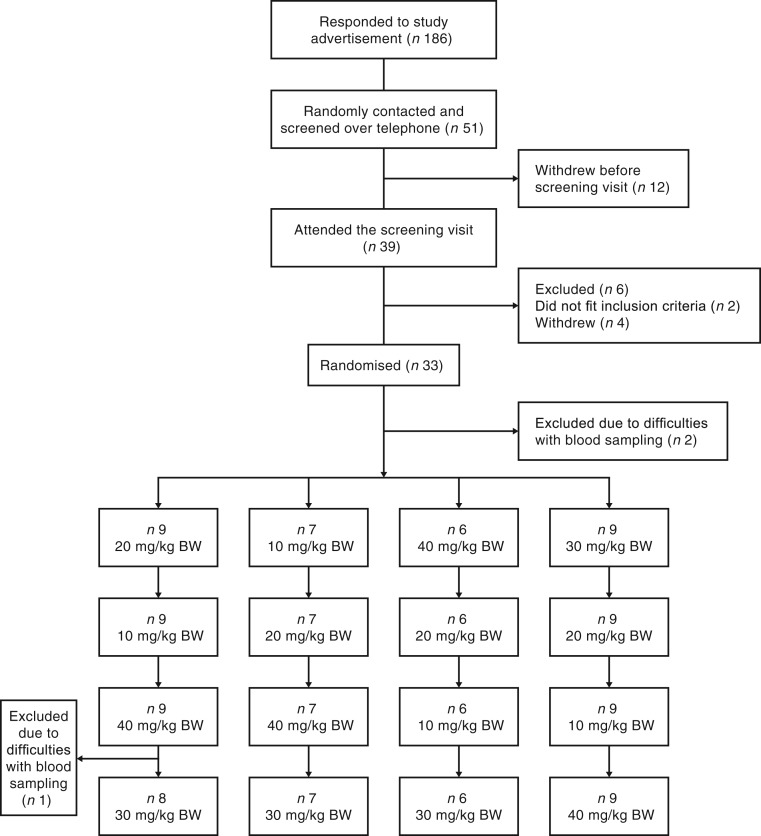
Flowchart depicting the inclusion and randomisation process. BW, body weight.

From April to June 2018 we screened fifty-one subjects for study participation and thirty-three were enrolled in the study (Fig. 1). Two of the included participants were excluded before the first test day due to difficulties with blood sampling. Overall, thirty-one subjects aged 60–78 years completed the trial (thirteen males and eighteen females). One participant had to be excluded on the final study day due to difficulties with blood sampling; therefore data on glucose, insulin and GLP-1 are only available for three of the dose levels. Four of the participants were excluded from the final statistical analysis of GLP-1 due to analytical errors. Baseline characteristics of the participants are presented in Table 2.

**Table 2.** Baseline characteristics of the thirty-one participants (Mean values and standard deviations; numbers of subjects)

	Mean	sd
Subjects (*n*)	31	
Male	13	
Female	18	
Age (years)	67·8	4·9
Weight (kg)	76·6	11·3
BMI (kg/m^2^)	26·0	2·6
Systolic BP (mmHg)	137	15
Diastolic BP (mmHg)	82	10
HbA1c (mmol/mol)	36·7	4·3
Fasting glucose (mmol/l)	5·4	0·6

BP, blood pressure; HbA1c, glycated Hb.

### Energy and macronutrient intake

No statistically significant differences were observed in energy intake or macronutrient intake during the course of the study (Table 3). One participant did not fill out the 3-d food record at the end of the study. Based on the reported intake of protein from the food diaries at baseline and at the end of the study, an average intake of 1·2 g protein/kg BW at baseline was estimated and this did not change during the study period (*P* = 0·36; estimated average intake at end of study 1·1 g protein/kg BW).

**Table 3.** Dietary intake at baseline and at the end of the study* (Mean values and standard deviations)

	Baseline		End of study		*P*†
	Mean	sd	Mean	sd	
Energy intake (kJ)	7765	1586	7807	1506	0·84
Protein (g)	86·9	20·6	84·2	18·1	0·32
Fat (g)	73·1	19·3	75·2	18·6	0·50
Carbohydrates (g)	215·2	65·4	213·8	55·8	0·95

* Food and drink intakes were registered for 3 d at baseline and at the end of the study.

† Paired-samples *t* tests were used to compare changes in energy intake and macronutrient intake from baseline to the end of the study. No significant differences were observed during the course of the study.

### Postprandial measurements

In a mixed-model analysis, no statistically significant differences in estimated maximum value of glucose, insulin or GLP-1 were observed when comparing the lowest dose of 10 mg/kg BW of CPH with 20, 30 or 40 mg/kg BW (Table 4). The estimated maximum value of glucose was on average 0·28 mmol/l lower when the participants were given the highest dose of 40 mg/kg BW CPH compared with the lowest dose of 10 mg/kg BW (*P* = 0·13). The estimated maximum value of insulin was on average 5·14 mIU/l lower after participants were given the highest dose of 40 mg/kg BW of CPH compared with the lowest dose of 10 mg/kg BW (*P* = 0·20). The estimated maximum value of GLP-1 was on average 0·34 pmol/l lower when given the highest dose (40 mg/kg BW) compared with the lowest dose of CPH (10 mg/kg BW) (*P* = 0·48). No carry-over effect was observed for glucose (*P* = 0·19), insulin (*P* = 0·21) or GLP-1 (*P* = 0·08).

**Table 4.** Estimated maximum values of glucose, insulin and glucagon-like peptide 1 (GLP-1) derived from a mixed model (Mean differences and 95 % confidence intervals)

Outcome	Dose level (mg/kg BW)	Mean difference	95 % CI	*P*
Glucose (mmol/l)	10	0	(Reference)	
	20	0·33	−0·04, 0·70	0·08
	30	0·09	−0·29, 0·46	0·65
	40	−0·28	−0·65, 0·08	0·13
Insulin (mIU/l)	10	0	(Reference)	
	20	3·59	−4·34, 11·5	0·38
	30	3·42	−4·60, 11·4	0·40
	40	−5·14	−13·1, 2·79	0·20
GLP-1 (pmol/l)	10	0	(Reference)	
	20	−0·66	−1·59, 0·28	0·17
	30	−0·11	−1·06, 0·84	0·83
	40	−0·34	−1·28, 0·59	0·48

BW, body weight.

**Fig. 2.**
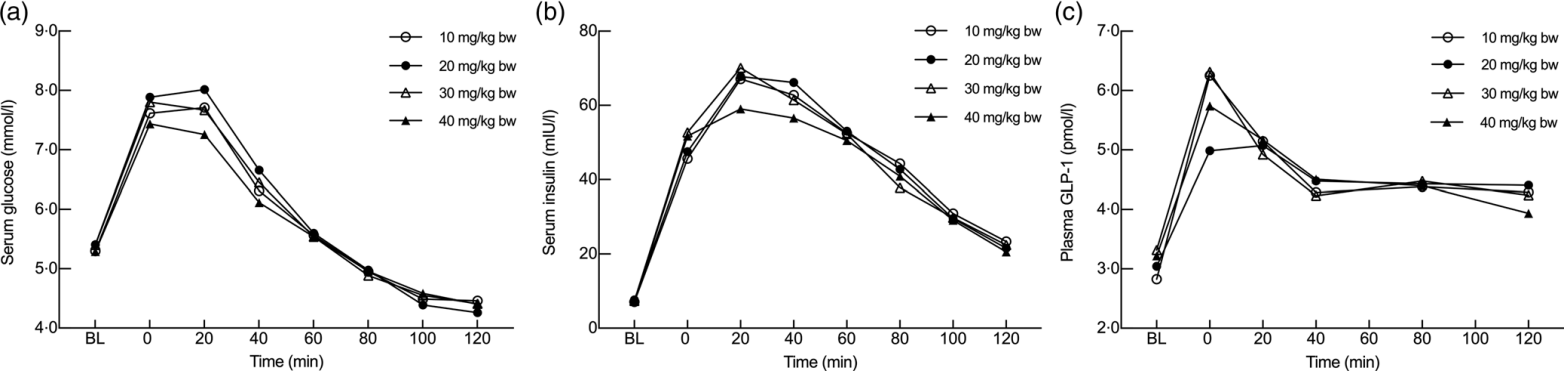
Metabolic responses for serum glucose (a), serum insulin (b) and plasma glucagon-like peptide 1 (GLP-1) (c) concentrations after intake of a standardised breakfast meal and the last dosage of the cod protein hydrolysate (CPH). Dose levels were 10, 20, 30 and 40 mg/kg body weight (BW). Results for serum glucose and insulin are presented for all thirty-one subjects, whereas for GLP-1 the results are presented for twenty-seven subjects (four participants were excluded from the statistical analysis due to analytical errors). Values are means. Time point 0 min is the first postprandial blood sample, taken 25 min after the drink was served and 15 min after the breakfast meal started. BL, baseline.

**Fig. 3.**
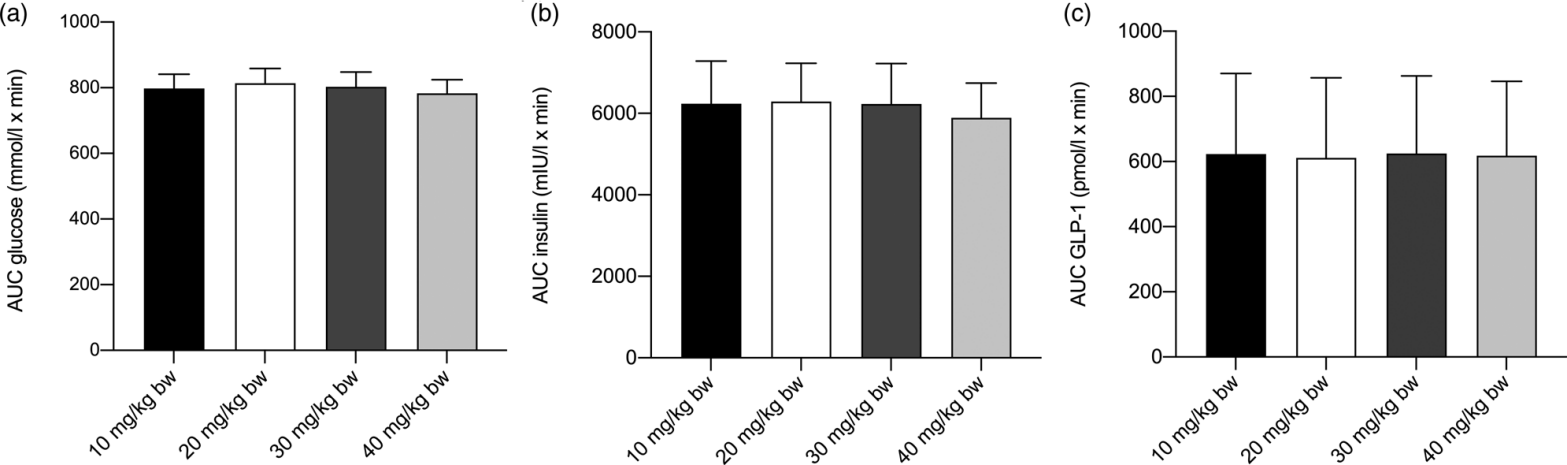
Bar chart depicting the total AUC for serum glucose (a), serum insulin (b) and plasma glucagon-like peptide 1 (GLP-1) (c) after intake of a standardised breakfast meal and the last dosage of cod protein hydrolysate (CPH) in the dose level. Dose levels were 10, 20, 30 and 40 mg/kg body weight (BW). Values are means, with standard errors represented by vertical bars. No statistically significant differences in AUC between the four different doses were observed for any of the outcome measures when comparing the lowest dose of 10 mg/kg BW of CPH with the higher doses of 20, 30 or 40 mg/kg BW.

No statistically significant differences in AUC between the four different doses were observed for any of the outcome measures when comparing the lowest dose of 10 mg/kg BW of CPH with the higher doses of 20, 30 or 40 mg/kg BW. For glucose, AUC was calculated from *t* = baseline until *t* = 80, excluding *t* = 100 and *t* = 120 (Fig. 2), based on the assumption that for the majority of individuals, glucose levels had returned to their baseline levels. The AUC for glucose was on average 1·16 mmol/l × min higher when given 20 mg/kg BW of CPH (*P* = 0·14), on average 0·27 mmol/l × min higher when given 30 mg/kg BW (*P* = 0·73) and on average 0·78 mmol/l × min lower when given 40 mg/kg BW (*P* = 0·32), when compared with the lowest dose of 10 mg/kg BW of CPH. If all measuring points were included in the statistical analysis of glucose, also including *t* = 100 and *t* = 120, the significance of the results did not change (Fig. 2). For insulin, the AUC was on average 11·3 mIU/l × min higher when given 20 mg/kg BW of CPH (*P* = 0·49), on average 6·84 mIU/l × min higher when given 30 mg/kg BW of CPH (*P* = 0·67) and on average 7·4 mIU/l × min lower when given 40 mg/kg BW (*P* = 0·65), when compared with the lowest dose of 10 mg/kg BW of CPH. For GLP-1, the AUC was on average 1·38 pmol/l × min lower when given 20 mg/kg BW of CPH (*P* = 0·36), on average 0·01 pmol/l × min lower when given 30 mg/kw BW of CPH (*P* = 0·99) and on average 1·09 pmol/l × min lower when given 40 mg/kg BW of CPH (*P* = 0·47), when compared with the lowest dose of 10 mg/kg BW of CPH. A graphical representation of the metabolic response for serum glucose, serum insulin and plasma GLP-1 concentration on the test day, the last day in the four different intervention cycles, is presented in Fig. 2. A bar chart showing total AUC for serum glucose, serum insulin and plasma GLP-1 is presented in Fig. 3.

### Adverse effects

No adverse effects were reported in the questionnaires, from the biochemical safety parameters or from the biochemical parameters for nutritional status.

## Discussion

The overall objective of the present study was to evaluate the effect of increasing doses of a supplement with CPH on glucose metabolism in older adults, aiming to find a dose response and creating a basis for an optimal daily dose for future clinical use. We investigated the effect on postprandial glucose regulation of four different doses of a CPH supplement (10, 20, 30 and 40 mg/kg BW) taken daily for 1 week. Although no statistically significant differences were observed between the postprandial measurements after the four different doses, our results indicate that the highest dose of CPH (40 mg/kg BW), equal to 3·2 g/d in an individual with a BW of 80 kg, is the most efficient in lowering postprandial blood glucose levels and insulin concentrations, when compared with the lower doses (10, 20 and 30 mg/kg BW).

In a previous publication, we reported that a single dose of 20 mg/kg BW CPH significantly reduced postprandial insulin concentrations in healthy, middle-aged to older individuals, without affecting postprandial glucose levels or GLP-1 levels, compared with control (casein)^(22)^. We hypothesised that the CPH might enhance the insulin sensitivity and affect other mechanism involved in blood glucose uptake in peripheral tissue. The significantly lower insulin concentration after intake of CPH may be of more interest in patients with reduced insulin sensitivity.

To our knowledge, only one small pilot study has been conducted with fish protein hydrolysate in an older population^(25)^. In this double-blind, randomised controlled study, a daily dietary supplement of 5·2 g fish protein hydrolysate from blue whiting, or placebo, was given to twenty-four nursing home residents daily for 6 weeks. No differences in serum concentrations of glucose or insulin after 6-week supplementation with fish protein were observed, when compared with placebo^(25)^. However, since this was a study population with older adults who lived in a nursing home setting, the results are not directly transferable to our study population with home-dwelling older adults.

Based on a few previous studies investigating the effect of supplements containing protein hydrolysates from fish on metabolic health^(17,26)^, we hypothesised that small doses of CPH may be effective due to the content of small, easily absorbable bioactive peptides. These are capable of rapidly affecting different metabolic pathways involved in glucose regulation and hence leading to a more rapid glucose response in the body. Thus, we presume that a potential observed effect on postprandial glucose metabolism can be attributed to the content of small, bioactive peptides in the supplement, and not the protein intake *per se*, which is negligible compared with overall protein content in a normal meal. Previous studies investigating supplements with fish protein or hydrolysates of fish proteins have reported doses in the range of 1 to 6 g per d to beneficially influence blood glucose metabolism when compared with control^(16,17,21,26)^.

The results have to be interpreted taking certain limitations in the design into account. The use of a cross-over design always implies a risk of a carry-over-effect. According to analysis of all possible interaction effects between doses and time periods, the results in this cross-over trial are not biased by a carry-over effect. We included a washout period of 7 d between each week of peptide supplementation. We presume 1 week to be a sufficient washout period, as dietary protein in general has a high turnover rate and the investigated doses of protein hydrolysate were low^(27)^. On study days, the supplement was given to the participants 10 min before breakfast and 25 min before the first postprandial blood sample was taken. This design might have caused a metabolic response even before the breakfast was served. As a result, we may have missed some early information on postprandial glucose response. Furthermore, a 2-week washout period for the use of supplements containing *n-*3 PUFA before starting on the first dose of CPH may not have been enough and a longer washout period could arguably have strengthened the design. It is possible that the short supplementation period of 1 week could have affected the results, and that a longer period would have been preferable. However, we have previously observed an effect after only one acute supplementation (20 mg/kg BW) in healthy middle-aged adults^(22)^. A longer intervention period would have made it more challenging to include participants and avoid drop-outs, due to a long time-frame of the study. Therefore, due to practical implementations of the study, 1 week of supplementation (7 d) for each dose was chosen. Finally, the design could have been strengthened by including a postprandial blood sampling at day 0 for each intervention cycle or a control group (0 mg/kg BW CPH). However the study was performed based on a previous study, where we report that a low dose of CPH (20 mg/kg BW) significantly reduced the postprandial insulin concentration^(22)^, and we therefore aimed to further evaluate the effect of different doses in the present study. An additional study day in each intervention period would also have made it more challenging to include participants and avoid a high drop-out rate, and would be difficult to implement due to limited resources. Based on this, we chose to only include postprandial blood sampling at the end of each intervention period.

To our knowledge, no previous publication has reported on the metabolic effect of different low doses of fish protein hydrolysate in an older adult population. Although no significant differences were observed in this trial, our findings suggest that low doses of fish protein hydrolysate might be effective and capable of improving blood glucose regulation in older adults. According to our findings, further studies investigating effects of supplements containing hydrolysates of fish proteins should be able to observe a metabolic effect from doses starting around 40 mg/kg BW, equal to 3·2 g per d in an individual with a BW of 80 kg. Based on this, we suggest that a dose ranging from 3 to 4 g per d is a reasonable starting point for future clinical studies. Due to preliminary findings, these results require further investigation.
